# Rein tension in harness trotters during on-track exercise

**DOI:** 10.3389/fvets.2022.987852

**Published:** 2022-10-11

**Authors:** Agneta Egenvall, Anna Byström, Mette Pökelmann, Malin Connysson, Kathrin Kienapfel-Henseleit, Magnus Karlsteen, Paul McGreevy, Elke Hartmann

**Affiliations:** ^1^Faculty of Veterinary Medicine and Animal Science, Department of Clinical Sciences, Swedish University of Agricultural Sciences, Uppsala, Sweden; ^2^Faculty of Veterinary Medicine and Animal Science, Department of Anatomy, Physiology and Biochemistry, Swedish University of Agricultural Sciences, Uppsala, Sweden; ^3^Wången, National Center for the Education and Development of Harness Racing and Icelandic Horse Riding, Alsen, Sweden; ^4^Agroscope, Swiss National Stud Farm, Avenches, Switzerland; ^5^Department of Applied Physics, Chalmers University of Technology, Göteborg, Sweden; ^6^School of Environmental and Rural Science, University of New England, Armidale, NSW, Australia; ^7^Faculty of Veterinary Medicine and Animal Science, Department of Animal Environment and Health, Swedish University of Agricultural Sciences, Uppsala, Sweden

**Keywords:** Standardbred, equine, welfare, racing, driving, rein tension

## Abstract

Horseracing is under public scrutiny with increasing demands to safeguard horse welfare. It is accepted that, as a result of bit pressure and/or equipment, mouth lesions accompany many types of horse use, including racing. However, there are currently no data available on the range of bit pressures in driven trotters. Our aim was to investigate whether rein tension (RT, proxy for bit pressures) differs among gaits, between tempo within gait, between horses and drivers, and between left/right reins. Standardbreds (*n* = 9), driven by experienced drivers (*n* = 11), performed exercise tests on a racetrack (cross-over design; total 31 tests, data available from 26 tests). Horses' motion symmetry was measured before tests (trotting in hand). Rein tension, speed and heart rate were measured during exercise. A moving-window filter was applied to RT raw data. Median, maximum and interquartile range for the estimated stride median RT were determined for each rein (left/right) and segment: walk; circling in slow trot followed by transition to faster trot; fast (racing) trot; and slowing down to walk. Mixed models were used for statistical analysis. Least square means for segment median RT ranged between 17–19 N in walk, 34–40 N during circling-accelerating, 51–62 N in fast trot, and 53–71 N for slowing down. Segment maximum RT was between 60–81 N in walk, 104–106 N during circling-accelerating, 72–86 N in fast trot, and 86–129 N during slowing down. Interquartile ranges were between 7–9 N in walk, 28–31 N during circling-accelerating, 8–10 N in fast trot, and 12–18 N for slowing down. Hind limb asymmetry exceeded the recommended threshold in three horses and was associated with higher median (48 N) and maximum (106 N) RT than symmetric horses (29 N and 73 N, respectively, *p* < 0.01). Consistent left-right asymmetry in RT was more common among horses than among drivers. Rein tension increased with increasing heart rate (p ≤ 0.0006). Rein tensions were higher than those reported during riding or in horses worked from the ground. The findings of high RT, taken together with the high reported prevalence of oral injuries in harness trotters, call for further research into RT, motion symmetry and use of equipment.

## Introduction

During horse handling and training, humans use cues to prompt horses to offer certain responses needed for particular tasks. When they reliably trigger one response only, these cues are labeled discriminative stimuli. Formerly referred to as “aids” (1), such stimuli can be auditory (e.g., vocalized cues), visual (e.g., the movement of a rope), or tactile (e.g., pressure applied to the horse's body). For riding horses, tactile cues arise from changes in the pressure from the riders' legs, their center of mass (also known as seat cues), and the bit, *via* rein tension. Additionally, the whip may be used to deliver cues. When the horse is driven, for example in harness racing (trotting), seat or leg cues are not applicable whereas bit cues represent an important avenue of communication between driver and horse. Of course, as in the ridden horse, the whip and the voice can also be used to communicate with the driven horse.

Rein tension has mainly been measured in riding horses during ridden work (2, 3), but also during handling horses from the ground (4, 5), long-reining (6), unridden exercise on the treadmill (7), and unridden and free-moving horses with side reins attached to the bit (8). Moreover, rein tension has been measured during carriage driving (9). To our knowledge, rein tension has not been measured in race-horses; neither in racing Thoroughbreds nor in Standardbreds or other horse breeds used for harness racing. In ridden horses, rein tension increases progressively across walk, trot and canter (2, 10, 11). Furthermore, extended gaits (higher speed within the same gait) are associated with higher tension than more collected (slower) variations of the same gait (12). When the rider is riding with shortened reins (with a so-called contact), there is a characteristic pattern of rein tension fluctuations that relates to the stride cycle. In general, there are one or two distinctive peaks through each stride, interleaved with longer periods of less rein tension. In trot, there is one peak during the suspension phase or early stance for each diagonal, coinciding with the downward motion of the horse's head relative to its body (12, 13) and upwards movement of the rider's body relative to the horse (14). Similar patterns have been observed in unridden horses when side reins were used (7). However, it is currently unknown whether these patterns persist at higher speeds with shorter stride duration (15).

Unfortunately, oral injuries are common in riding horses (16–19) specifically when examined after a competition. For example, 10–52% of competition horses had signs of acute oral lesions in the area of the bit (20, 21). In harness racing, the frequency of acute oral lesions after competition including Standardbreds, Finnish coldblooded trotters and ponies was even higher, 84% (22). This raises questions regarding horse welfare and the effectiveness of bit cues. This pertains particularly to driving because drivers have fewer types of discriminative stimuli to apply than riders. During harness racing, where several horses are running at high speed in a tight field, misunderstandings between driver and horse can be very dangerous, e.g., the horse breaking into gallop or not slowing down or stopping when cued (e.g., during line-up for the start). Furthermore, even slowing down considerably during the race may represent a safety risk. Thus, forwardness (the tendency to accelerate rather than decelerate) is a prerequisite for both safety and for performance during harness racing. Drivers generally consider it desirable that trotters accept (and therefore largely ignore) a firm bit pressure at racing speed, such that the main cue for acceleration during racing is to release the rein tension. This suggests that bit pressures/rein tension may be high during harness racing.

The aim of the current study was to quantify rein tension in Standardbred trotters during walk and trot over a range of speeds and events in an exercise test designed to reflect situations common during racetrack training. It was hypothesized that the tension would differ between gaits, between different speeds in trot, and during different events of the exercise test, such as transitions between gaits, or trotting fast in the direction that horses normally compete in (counter-clockwise direction) compared to the opposite direction (clockwise direction). It was further hypothesized that rein tension would differ among horses, among drivers and between left and right rein. Variables related to driver and horse characteristics, e.g., age and experience, were also evaluated as possible predictors of rein tension.

## Materials and methods

The study was carried out at Wången, the National Center for the education and development of harness racing and Icelandic horse riding in October 2021. It was designed to measure the magnitudes of rein tension in Standardbreds driven on the racetrack, and to investigate drivers' experiences of the behavior and driveability of each horse they drove. The latter data are presented in detail in (23). The methods used in this study were approved under the protocol A 18-2021 by the Umeå Local Animal Ethics Committee.

### Horses and management

Nine race-trained Standardbred trotters were recruited for this study, kept for at least 6 months (eight horses) and 1 month (one horse) at Wången. There were four mares and five geldings, between 3 to 14 years (median 6 years) with bodyweights ranging from 413 to 597 kg (median 497.2 kg). The horses were regularly driven by students under the supervision of staff members and licensed trainers (Swedish Trotting Association). Seven of the horses had participated in official races (number of races 24.8 ± 8.5) with average lifetime earnings of 116 096 SEK ± 35 088.

Two of the horses were kept in a 24-h loose housing system with automatized feed provision and free access to straw. The remaining seven horses were stabled in individual boxes (3 x 3 m, wood shavings) during nights and were turned turned-out for 4–6 h in pairs or groups during daytime. All horses received daily rations between 11 and 13 kg of haylage and concentrated pelleted feed (consisting of 1.4 kg ± 0.3, Krafft Groov Original, Lantmännen Krafft AB, Sweden). Water was offered ad libitum.

One day prior to the start of the experiment, general health and locomotory soundness were evaluated by Wången's equine veterinarian. A simple mouth check, without sedation or mouth gag, was also performed. This included palpation of the external and buccal commissures of the upper and lower lips and a visual examination of accessible buccal areas including the mandibular diastema and tongue. All horses were considered free of bruises, lesions or wounds in the mouth.

### Drivers

A total of 11 drivers (7 females, 4 males) were enrolled in this study. Drivers were asked to complete a questionnaire (23) detailing their age, experience with horses and driving, as well as their handedness (whether using the right or left hand for writing, throwing, brushing teeth, cutting and eating with a spoon). Analysis revealed that ten of the eleven drivers were right-handed.

Seven of these drivers were students (17–18 years old) at Wången, of whom all were sufficiently experienced in race-track driving of Standardbreds. The students had on average 8.6 ± 1.9 years of driving experience. Five of the students had competed in official harness races (number of races 34.2 ± 14.2). The remaining four drivers were staff members, two female and two male, with prior experience as licensed drivers or trainers. Their age ranged between 29 and 65 years (46.5 ± 7.4) and their driving experience was on average 31.3 ± 10.1 years. Two of the drivers had competed in 4 up to 50 races, and one staff member in over 10.000 races.

### Driving equipment

The horses were prepared in the aisle of their home stable, wearing their regular training equipment, i.e., open bridles with loose nosebands and single-jointed metallic driving bits. Overcheck bits or any other equipment restricting head/neck/mouth movements were not used. A Grafström Speedcart Sport Classic training cart was used for all horses except for one horse that was driven in a Finntack T4 Speedcart. Stainless steel reinforced, synthetic or leather driving reins (weight 1.1–1.2 kg) were used.

### Exercise test

A predefined exercise was performed by drivers and horses at Wången's racetrack (a 1,000 m long oval banked gravel track) in a partial cross-over design, over four consecutive days. The exercise protocol consisted of walk and trot in both directions. After a warm-up in moderate trotting speed (jog), horses were walked to the finish line, circled in slow trot (20 m in diameter), then driven straight along the track accelerating up to racing speed, with the speed being maintained for three quarters of a lap around the track (two curves and one long side), before being slowed down to walk again. This was repeated twice, first circling to the right and racing in left direction around the track, then circling left and racing right (see Table 1).

**Table 1:** Exercise protocol showing the segments studied while nine horses where driven by 11 different drivers (total 31 test drives) on an oval banked racetrack in both clockwise (right) and counter-clockwise (left) direction.

**Segment description**	**Segment**	**Gait**	**Direction**	**Distance**	**Speed (m/s)**		**HR (bpm)**	
	**acronym**			**(m)**	**Mean**	**SD**	**Mean**	**SD**
Walking from stable to racetrack^*^		Walk		50–100	1.6	0.2	77	12.8
Warming-up in slow trot^*^		Trot	Right	1,000	5.0	0.6	110	18.0
Walking from finish line to circle	WalkFLCircle	Walk	Right	120	2.2	0.3	106	15.1
4 circles in trot (Ø 20 m)	CircStartLeft	Trot	Right		5.5	0.6	131	24.2
Racing (fast) trot	RacingRight	Trot	Left	200	10.4	0.4	175	42.8
Racing (fast) trot and slowing	SlowingRight	Trot	Left		8.8	1.2	177	37.3
Transition to slow trot and walking to finish line	Trans2WRight	Walk	Left	120	2.1	0.3	132	10.3
4 circles in trot (Ø 20 m)	CircStartRight	Trot	Left		5.6	0.7	156	13.0
Racing (fast) trot	RacingLeft	Trot	Right	200	10.9	0.5	196	23.4
Racing (fast) trot and slowing	SlowingLeft	Trot	Right		8.7	1.5	189	21.5
Transition to slow trot, walking to finish line and back to stable	Trans2WLeft	Walk	Right	120	2.0	0.2	134	9.1

The order of segments presented in the table corresponds to the order in which they were performed. Speed (m/s) and heart rate (HR, beats per minute) were recorded for each horse and exercise test.

* Segment not included in statistical analysis.

On each of the four data collection days, the horses were tested in groups of three horses. This was done to avoid separation anxiety from conspecifics but also to mimic race training in a field of horses. Each horse was tested only during 1 day except for two horses that were driven again on the last of the 4 test days. During the latter, to assure the same test conditions throughout all exercise tests, they were joined by a third horse from which no measurements were taken. During a test day, each horse was driven in three repetitions of the same test, and two of the horses were driven twice during their second test day. Thus, either three or two horses, as well as drivers, participated each test day, and each driver drove each horse once. This resulted in a total of 31 trials. Allocation of drivers to horses was done randomly. One researcher manually noted the times at which the first horse passed selected locations on the racetrack that were associated with changes in speed and direction.

Apart from warming-up, the horses were driven on the track in the following constellation: one horse in leading position, the second horse outside and slightly behind the leading horse, and the third horse right behind the leading horse on the inside of the racetrack. Horse positions were alternated between repetitions, such that each horse performed each test in a different position. However, the drivers retained their positions during the tests, i.e., the driver drove all horses in the same position, for example always in leading position.

### Measuring equipment

Prior to their first exercise test, motion asymmetry of the head and croup was examined once in all horses using an inertial measurement unit (IMU)-based gait analysis system (Equinosis, Columbia, MO, USA). One sensor with a uni-axial gyroscope (measuring range 0–300°/s) was attached to the right forelimb pastern with a specially designed pastern wrap. Sensors with three uni-axial accelerometers (measuring range 0–6 G) were positioned at the poll (the atlanto-occipital junction) using a felt poll bumper, and at the midline of the dorsum between the two sacral tuberosities using double-adhesive tape. The sensors measured 3.2 x 3.0 x 2.0 cm and weighed 28 g. Data were recorded at 200 Hz (8-bit resolution) and wirelessly transmitted to a handheld computer. The horses were evaluated during trot in a straight line on firm ground (in the stable aisle), until a minimum of 15 strides had been recorded. The handler was leading the horses on a loose lead rope from their left-hand side to not interfere with their head carriage.

Left and right rein tension was measured with load cell sensors (IPOS Technology B.V., 5656 AE Eindhoven, The Netherlands, https://www.ipostechnology.com/). The sensors (weight 68 g) were placed between the bit and the rein using a leather shaft strap. One additional leather strap was attached between the bit and rein for safety reasons in case the IPOS sensor broke. The measuring range was stated by the manufacturer to be between 0 and 500 N. Data were sampled at a rate of 80 Hz. Measurements were received wirelessly through a smartphone carried by the drivers (through the IPOS app) *via* Bluetooth (minimum required version 4.3). Using the app, rein sensors were calibrated (zero offset adjusted) before each test, placing the sensors on a horizontal surface with no applied load. The calibration was checked by using five known reference weights ranging from 1.25 to 15 kg. The readings indicated good accuracy within this measuring range (data not presented).

Speed and heart rate were measured during each test drive at 1 Hz using the Polar Heart Rate Meter M460 together with a Polar H10 heart rate sensor (Polar Electro, Kempele, Finland). Data were downloaded to PolarFlow (https://flow.polar.com/) and exported to Excel. A GoPro camera (HERO 8, GoPro, Inc., San Mateo, USA) was mounted on the helmet of each driver to enable video documentation of the test drives.

### Data management and missing data

The data from the gait analysis system collected before exercise tests were processed by the gait analysis proprietary software, which analyses the data automatically. Recorded vertical accelerations are converted to vertical displacement using a moving-window, error correcting, and double integration algorithm (24). These data were split into strides, based on sagittal plane angular velocity data from the limb-mounted sensor's gyroscope. Stride-by-stride difference in vertical displacement minima and maxima for the poll (HDmin, HDmax) and pelvis (PDmin, PDmax) between right and left limb stances were calculated and reported together with trial means and standard deviations (SD). Horses were classified as asymmetric if mean PDmin or PDmax values exceeded 3 mm for the hind limbs, and their SD was lower than the values, and if HDmin exceeded 6 mm for the forelimbs and the SD was lower than the mean value.

The IPOS app automatically uploaded the recorded data to a cloud database. Raw rein tension data were then downloaded *via* a web-interface available via the IPOS website (https://www.ipostechnology.com/). The data were further handled in Matlab (version R2020a, Mathworks, Natick, MA, USA).

**Figure 1:**
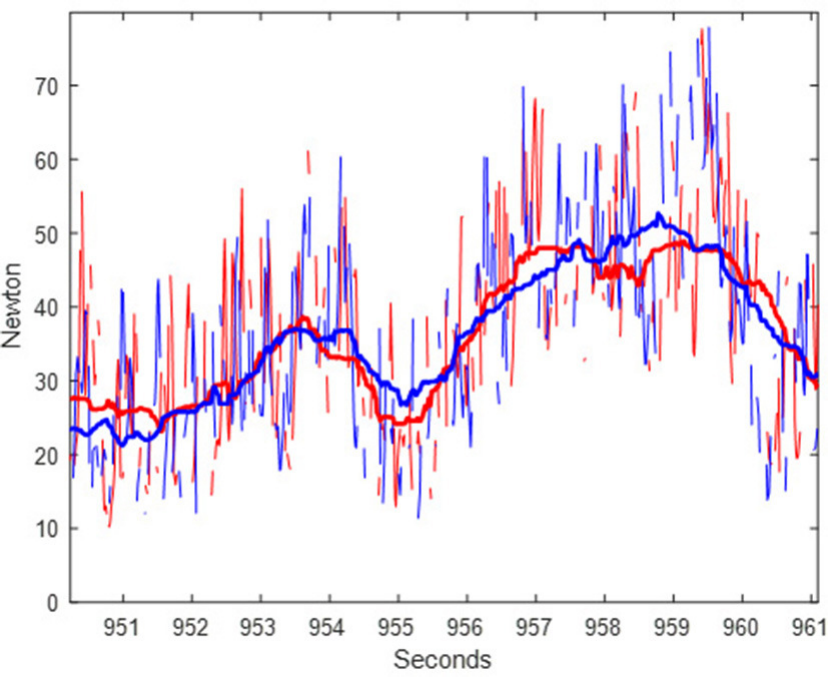
Example of raw and filtered rein tension data from a segment of racing (fast) trot for one horse during 10 s. Traces represent raw and filtered data for the left (red) and right (blue) rein.

**Figure 2:**
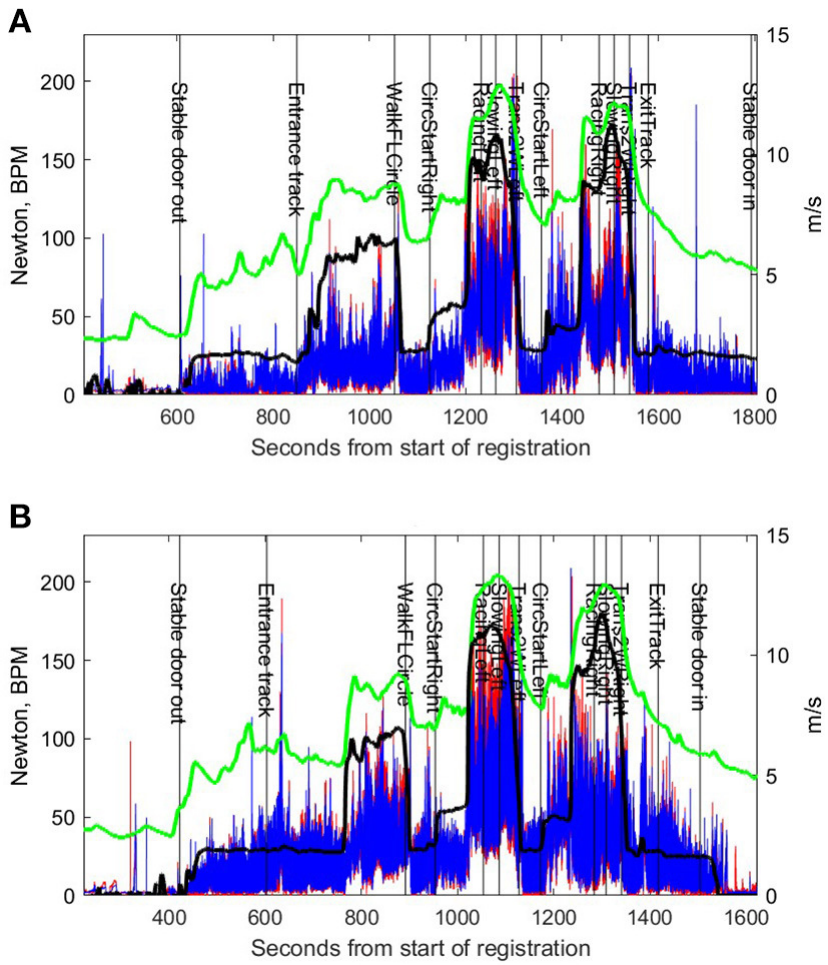
Rein tension, speed and heart rate data from the same horse driven by two different drivers, **(A,B)**. Red (left rein) and blue (right rein) traces represent median-filtered rein tension. The black trace represents speed (m/s) and the green trace represents heart rate (beats per minute). The vertical lines and acronyms indicate start of each the exercise test segment (see Table 1) based on the manual time protocols.

Initial data scrutiny revealed that many trials had considerable data loss. In five trials, the data registration had been aborted almost directly after the start of the trial, so the data from these trials had to be discarded completely. In one trial, data logging had stopped halfway into the trial, hence this trial lacked data for the second half. Of the remaining 25 trials, many had a large number of missing values, mainly dispersed throughout the measurements, though periods with continuous data loss for up to 30 s also occurred in about half of the measurements. Among the measurements with useable data, the percentages of minimum, median and maximum data loss were 1, 16, and 49%, and 1, 14 and 25%, for left and right rein tension, respectively (based on the expected number of samples, calculated from measurement duration in seconds and an 80 Hz sampling rate, as stated by the manufacturer). Because of this considerable data loss, it was not possible to accurately analyze stride-associated variations in rein tension. Thus, further analysis focused instead on estimating stride median rein tension. As gait events, e.g., hoof-on times, were not available, stride median rein tension was approximated by applying a moving median filter with a fixed window width of 1.4 s, corresponding to at least one stride at walk and two strides at fast trot. Figure 1 demonstrates how raw and filtered data compare in a short sequence of fast trot, to illustrate the effect of the filter. The median-filtered data for each trial were split into segments using the clock-times recorded during the experiment. Data outtake is illustrated in Figure 2, in which segment start and end times are indicated as vertical lines. To account for these times being recorded only for the first horse in the field, 3 s of data were excluded at the start and end of each segment in all tests. Data for each segment were visually inspected in time series plots and as box plots. Median, maximum and interquartile range per rein (left/right), segment, horse and driver were calculated from the median-filtered rein tension data (approximated stride median rein tension values) to be used in the statistical analysis.

Speed and heart rate data from the Polar system were manually synchronized with the rein tension data, by overlaying rein tension with speed and heart rate curves. Speed and heart rate data were then used as a guide to correct mistakes in the manual protocols where necessary, for example, if start or stop times were missing for some segments. Speed and heart rate data were then also split into segments and, as for rein tension, data from 3 s at start and 3 s at end of each segment were excluded. Mean and standard deviations for speed and heart rate were calculated per segment.

### Statistical analysis

SAS (version 9.4, SAS Institute Inc., Cary, NC, US) was used for statistical analysis. Mixed models (SAS-procedure MIXED) were used to analyse the outcome parameters segment median, segment maximum and segment interquartile range for the left and the right rein. Data for the following segments (see Table 1) were included: WalkFLCircle, CircleStartRight, RacingLeft, SlowingLeft, Trans2Wleft, CircleStartLeft, RacingRight, SlowingRight and Trans2WRight. Normality of outcome parameters was investigated using Box-Cox transformation (SAS-procedure TRANSREG) and visualized using QQ-plots. Based on this, median rein tension and interquartile range were analyzed as the fourth root, and maximum rein tension as the square root of the original data.

All models, the full models as well as the final, reduced models, included the following random effects: horse, driver, horse × driver and left/right rein × horse. Design variables were forced as fixed effects, i.e., segment, repetition number each day, and position in the field during the test. Rein (left/right) was initially tested as a fixed effect but was non-significant in all analyses. Horse sex, driver sex, driver years of experience (<10 years or ≥10 years), whether the driver had participated in official races (yes/no), and whether the horse was forelimb or hind limb asymmetric above the lameness locator threshold (yes/no for each pair of limbs), were all used as categorical effects. Additionally, horse age and segment median heart rate were included as linear effects, after verifying reasonable linearity vs. outcome parameters through plotting. All categorical and linear effects were tested one by one upon a model with the forced (fixed) design variables and the random effects. Significant effects were then combined in one model per outcome variable, also including the forced design variables. These models were then reduced. During preliminary modeling, the interaction between segment and position in the field was also tested.

To investigate if there were significant differences between drivers or horses, models with segment median rein tension as outcome were made with driver or horse as a fixed effect, while removing the same variable from the random part. That is, if driver was used as fixed effect, then driver and driver × horse were removed from the random part (compared to the models described above). Along with horse or driver, the same forced fixed effects as described above were included in each model. To investigate individual symmetry in rein tension, driver-specific models (including all horses that a driver drove) and horse-specific models (including all drivers that drove any given horse) were made to estimate within-subject differences between median left and right rein tension. These models contained only one random effect, horse in the driver-specific models and driver in the horse-specific models, while the fixed effects were rein, segment, repetition number and position in the field.

Least square means were calculated for each model and back-transformed for easier interpretation (both formats presented). The SAS-option PDIFF was used for pair-wise comparisons of categories when the type III *p*-value was significant. The *p*-value limit was set to 0.05. For each model, the contributions of the different random factors were quantified as a percentage of the total random variation.

## Results

### General results

The final dataset comprised data from nine horses driven by 11 drivers. There were 26 trials (6 of 31 trials recorded were excluded due to rein sensor malfunction), and for one trial, data were only available for the first seven segments. Based on the asymmetry measurements conducted prior to the exercise tests, one horse had forelimb asymmetry, two horses had hind limb asymmetry, and one horse was both fore- and hind limb asymmetric according to the lameness locator thresholds (Supplementary Table 1). Table 1 shows the distributions of mean speed and heart rate per segment.

**Figure 3:**
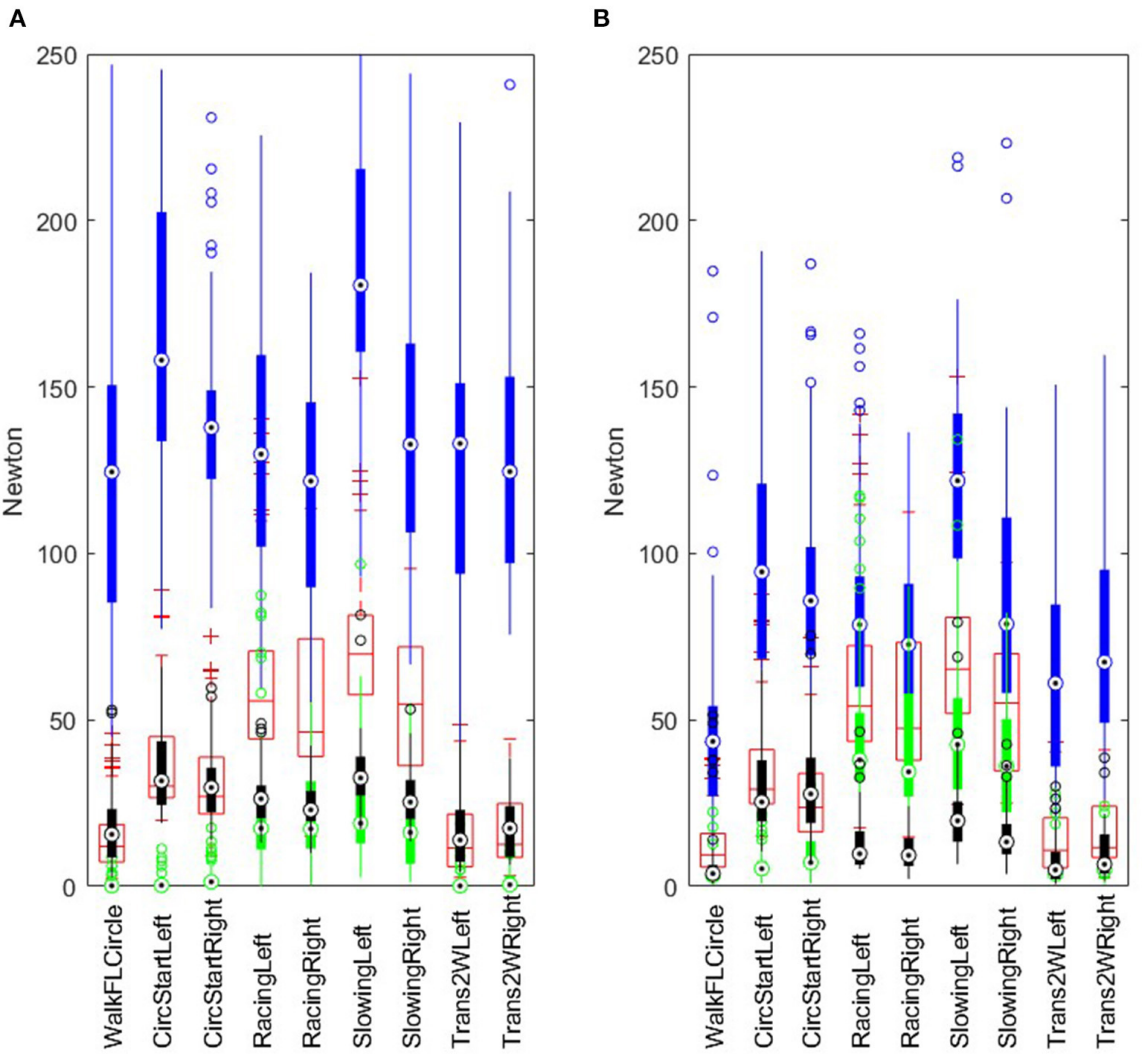
Boxplot of segment minimum (green), median (red), maximum (blue) and interquartile range (black) for raw **(A)** and filtered **(B)** rein tension, including data for both left and right reins. Data from 26 horse-driver combinations (*n* = 26 or 25 for each segment, as there was partial data loss for one measurement). For explanation of segment acronyms, see Table 1.

Figure 1 shows an example of raw and median-filtered data for the same time sequence, illustrating the effect of the filter. The median-filtered data for left and right reins are represented through the lines in the middle of the raw data; the raw data having a considerably larger range (variation within and between strides). Figure 3 demonstrates the distributions of minimum, median, maximum and inter-quartile range rein tension in raw and median-filtered data, respectively, for each segment, combining left and right rein tension data. This figure shows that the maximum peaks from the raw data are indeed different from those in the filtered data, which is expected as the filtered data are intended to represent the stride median. Segment medians are similar between raw and median-filtered data.

The interaction between segment and position in the field did not contribute to the median rein tension model. It was significant in the interquartile range model but with a larger Akaike criterion value (suggesting that the model was not better in explaining the data). However, in the maximum rein tension model, the interaction between segment and position in the field was significant (*p* = 0.0009). For example, there were 28 comparisons between positions within segments and, of these five were significant; four of which involved walk segments and one of which showed a difference in rein tension between the second (108 N) and third position (151 N) while racing in clockwise direction (*p* = 0.003). For ease of interpretation, we have presented results without this interaction.

### Model results for median rein tension

In the model with segment median rein tension as the outcome, significant variables were segment, hind limb asymmetry above the lameness locator threshold (yes/no) and heart rate (Table 2). Estimated least square means for segment median rein tension were between 17 and 19 N in walk, 51–62 N in fast trot (racing), 34–40 N during the start phase (circling at slow trot, then accelerating toward racing speed) and 53–71 N for slowing down. Of 36 between-segment comparisons, 32 were significant (*p* < 0.05, 26 of these 32 comparisons with *p* < 0.0001), indicating clear differences in median rein tension between most segments. The non-significant comparisons were between RacingRight and SlowingRight, and three comparisons between walk segments. Position in the field was non-significant (*p* = 0.97). Hind limb asymmetry was associated with almost twice the magnitude of rein tension compared to being symmetric (least square means 48 N vs. 28 N, *p* < 0.01). Rein tension increased with increasing heart rate (*p* < 0.0001). On the transformed scale, the estimated increase per BPM (beats per minute) was 0.0030 (SE 0.0005). This suggests that if, for example, the heart rate increased from 100 to 200 BPM, median rein tension would increase from 16 to 28 N.

**Table 2:** Least square means (478 observations, 11 drivers and 9 horses) from model results with segment median rein tension as the outcome variable (modeled fourth-root transformed) and *p*-values for the corresponding main effects.

		**Least square means**			**Type III**
**Variable**	**Category**	**Est**	**SE**	**Back-trans**	***p*-value**
Segment	WalkFLCircle	2.05	0.08	17.8	<0.0001
	CircStartLeft	2.52	0.08	40.5	
	CircStartRight	2.41	0.08	33.6	
	RacingLeft	2.81	0.08	62.4	
	RacingRight	2.67	0.08	50.6	
	SlowingLeft	2.90	0.08	70.9	
	SlowingRight	2.70	0.08	53.3	
	Trans2WLeft	2.04	0.08	17.3	
	Trans2WRight	2.08	0.08	18.8	
Repetition	1	2.54	0.08	41.7	0.07
number	2	2.45	0.08	36.1	
	3	2.41	0.08	33.5	
Position	1	2.47	0.09	37.4	0.97
In the field	2	2.45	0.10	36.1	
	3	2.47	0.09	37.3	
Hind limb	No	2.30	0.08	27.8	0.01
Asymmetry	Yes	2.63	0.12	48.1	

The variables segment (gait and direction), repetition number (first, second and third test drive) and position in the field (1-first, 2-second, and 3-last) were forced. For explanation of segment acronyms, see Table 1.

Est- estimate, SE-standard error, Back-trans- back transformed.

### Model results for maximum median rein tension

With segment maximum for the median-filtered rein tension as outcome, segment, hind limb asymmetry and heart rate were significant (Table 3). Estimated least square means were between 60–81 N in walk, 72–86 N in fast trot (racing), 104–106 N during the start phase (circling then accelerating) and 86–129 N during slowing down. Of the 36 between-segment comparisons, 29 were significant (*p* < 0.05, 16 of these with *p* < 0.0001). Of the seven non-significant comparisons, five were between walk and trot segments and two between trot segments. Both repetition number and position in the field were non-significant. Hind limb asymmetry above the lameness locator threshold was associated with increased maximum rein tension (106 N) compared to being symmetric (73 N, *p* = 0.003). Segment maximum rein tension increased with increasing heart rate (*p* = 0.0006). On the transformed scale, the estimate was 0.010 (SE 0.004). This suggests that if the heart rate increased from 100 to 200 BPM, for example, segment maximum rein tension increased from 81 to 101 N.

**Table 3:** Least square means (478 observations, 11 drivers and 9 horses) from model results with segment maximum rein tension as the outcome variable (modeled square-root transformed) and p-values for the corresponding main effects.

		**Least square means**			**Type III**
**Variable**	**Category**	**Est**	**SE**	**Back-trans**	***p*-value**
Segment	WalkFLCircle	7.72	0.37	59.7	<0.0001
	CircStartLeft	10.30	0.33	106.1	
	CircStartRight	10.19	0.34	103.9	
	CircStartLeft	9.28	0.34	86.1	
	CircStartRight	8.51	0.37	72.4	
	SlowingLeft	11.36	0.34	129.1	
	SlowingRight	9.28	0.36	86.0	
	Trans2WLeft	8.33	0.34	69.4	
	Trans2WRight	8.98	0.34	80.7	
Repetition	1	9.74	0.38	94.8	0.17
Number	2	9.01	0.36	81.1	
	3	9.24	0.35	85.4	
Position	1	9.28	0.36	86.1	0.14
In the field	2	8.94	0.39	79.9	
	3	9.77	0.34	95.4	
Hind limb	No	8.45	0.28	71.4	0.002
Asymmetry	Yes	10.21	0.47	104.2	

The variables segment (gait and direction), repetition number (first, second and third test drive) and position in the field (1-first, 2-second, and 3-last) were forced. For explanation of segment acronyms, see Table 1.

Est- estimate, SE-standard error, Back-trans- back transformed.

### Model results for interquartile range rein tension

In the model with interquartile range rein tension as outcome, segment and heart rate were significant (Table 4). Estimated least square means for segment interquartile range were between 7–9 N in walk, 8–10 N in fast trot (racing), 28–31 N during the start phase (circling then accelerating) and 12–18 N for slowing down (Table 4). Of the 36 between-segment comparisons, 28 were significant (*p* < 0.05, 20 of these with *p* < 0.0001), with non-significant comparisons being distributed across segments without a clear pattern. Rein tension interquartile range increased with increasing heart rate (*p* < 0.0001). On the transformed scale, the estimate was 0.0029 (SE 0.0006). This suggests that if, for example, heart rate increased from 100 to 200 BPM, the interquartile range for rein tension increased from 5 to 9 N.

**Table 4:** Least square means (478 observations, 11 drivers and 9 horses) from model results with segment interquartile range rein tension as the outcome variable (modeled fourth-root transformed) and *p*-values for the corresponding main effects.

		**Least square means**			**Type III**
**Variable**	**Category**	**Est**	**SE**	**Back-trans**	***p*-value**
Segment	WalkFLCircle	1.61	0.07	6.8	<0.0001
	CircStartLeft	2.30	0.06	27.8	
	CircStartRight	2.35	0.06	30.7	
	RacingLeft	1.79	0.06	10.3	
	RacingRight	1.65	0.06	7.5	
	SlowingLeft	2.06	0.06	17.8	
	SlowingRight	1.85	0.06	11.6	
	Trans2WLeft	1.62	0.06	6.9	
	Trans2WRight	1.73	0.06	9.0	
Repetition	1	1.95	0.06	14.5	0.19
Number	2	1.86	0.06	12.0	
	3	1.84	0.06	11.5	
Position	1	1.90	0.06	13.0	0.33
In the field	2	1.83	0.06	11.2	
	3	1.93	0.06	13.7	

The variables segment (gait and direction), repetition number (first, second and third test drive) and position in the field (1-first, 2-second, and 3-last) were forced. For explanation of segment acronyms, see Table 1.

Est- estimate, SE-standard error, Back-trans- back transformed.

### Random variation contribution by horse, driver, and driver and horse combination

Horse × rein (left/right) contributed with 1% of the total random variation in the model with segment median rein tension as outcome, 1% in the model for maximum and 0% in the interquartile range model. Horse similarly contributed with 27, 8, and 14% to the random variation, driver 13, 0, and 0%, and driver × horse with 9, 19, and 14% in median, maximum and interquartile range models, respectively. The residual, unexplained variation constituted 49, 72, and 72%, respectively, in the same three models.

### Differences between drivers and horses

In the segment median rein tension model with driver as fixed effect and horse as random, the overall effect of driver was significant (*p* < 0.0001). Of the 55 pairwise comparisons between drivers, 19 were significant (*p* < 0.05) and, of those, four were strongly significant at *p* < 0.0001 (see Supplementary Figure 1).

In the median rein tension model with horse as fixed effect and driver as random, the overall effect of horse was significant (*p* < 0.0001). Of the 36 pairwise comparisons between horses, 23 were significant (*p* < 0.05) and, of those, 11 were strongly significant at *p* < 0.0001 (see Supplementary Figure 1).

### Driver and horse-specific differences between left and right rein tension

While none of the drivers showed significant differences between left and right rein tension, four of the horses showed significant left-right differences. Of those horses, three showed significantly higher tension in the left rein and one in the right rein. Two of these four horses were from the group labeled as asymmetric according to the lameness locator threshold values. Of the two asymmetric horses, one was both fore and hind limb asymmetric and one was only hind limb asymmetric (see Supplementary Figure 1).

## Discussion

### Interpretation of the data

Given the unexpected problems with rein tension data loss, data analysis in the current study had to be adapted accordingly. Therefore, analysis of stride-related patterns, including stride maximum and range, was not attempted. Instead, stride median rein tension was estimated using a moving-window median filter, and differences in estimated median rein tension between different segments of the exercise test were investigated. Figure 1 illustrates that while the stride cycle related pattern is practically eliminated in the filtered data, variation over time, across multiple strides, followed a similar pattern compared to the raw data. Figure 3 further illustrates that the differences between segments followed a comparable pattern between raw and filtered data. However, maximum values were notably lower, and interquartile ranges narrower for the filtered data than for the raw data. This is to be expected, given that the stride-related fluctuations were filtered out. Rein tension data are otherwise seldom filtered before calculating discrete point variables for statistical analysis (11). Accordingly, median rein tension from the current study can be compared to median (and perhaps mean) rein tension in other studies, but (segment) maximum values cannot be directly compared. Despite these limitations, the results still provide an indication of levels of rein tension in trotters, which is valuable given that very little information was available prior to this study.

Based on raw data graphs from the measurements with less data loss (data not shown), it seems that rein tension in trotters displays a stride pattern similar to that seen in riding horses at the trot (12), with one peak per step (diagonal/half-stride) even at racing speed. In riding horses at the trot, the rein tension peaks during the suspension phase and is the lowest at midstance (12). Based on the similarity in patterns, it is reasonable to assume that the same is true for trotters, though this could not be confirmed because no temporal data (e.g., hoof ground contact times) were recorded in the current study.

### Levels of rein tension in trotters vs. riding horses

An early report on rein tension in racing Standardbreds describes peak tensions of up to 392 N (9). In the current study, segment maximum rein tension values determined from the raw data showed a median across all measurements that was above 100 N for all segments, above 150 N for two segments (RacingLeft, SlowingRight), and above 250 N in some measurements (Figure 3A). Segment median rein tension estimated from median filtered data (least square means) ranged between 34 and 71 N for the various trot segments. These figures are all considerably higher than values reported for riding horses at trot (10). That said, the levels of rein tension in riding horses do appear to increase with increasing speed. Extended trot in riding horses was associated with a median rein tension of 29 N (12), slightly higher than at sitting canter (25), while tensions for sitting and posting (rising) trot were lower, between 14 to 19 N per rein (2, 12). Riding horses typically trot at speeds ranging between 3 and 5 m/s while racing trot speed in the current study was around 10 m/s. This may explain the differences in rein tension levels, even though it is not entirely clear why higher speeds are associated with higher levels of rein tension or how this repeated finding may be unpacked in future studies.

During riding, the rider is subjected to higher forces at higher speed (26), likely a consequence of maximum reaction forces between the horse and the ground increasing exponentially with increasing trotting speed (27). As the speed increases, this makes it more challenging for the rider to maintain a stable position and to hold the hands a constant distance from the horse's mouth. As the ground reaction forces of the horse are transmitted to the sulky, the same also pertain to a driver, at least to some extent. In addition, for riders and drivers alike, increasing speed presents an increasing challenge to maintain control over the horse's speed and direction. Perhaps the higher speed in racing Standardbreds increases the need to have a firm contact for safety reasons, this may include anchoring the driver to the sulky. Furthermore, the partial release of rein tension as a signal for speeding-up is also a likely reason for the higher rein tension in general in trotters. It is expected that any future study of gallopers would reveal a similar trend. Median rein tension in walk observed in the current study (below 20 N) was reasonably close to median rein tension in walk reported in riding horses. Eisersiö et al. (2) found median rein tension of 12 N per rein in horses walking on short reins.

High rein tension may be associated with oral injuries in the area of the bit, and such lesions are indeed frequent in trotters after racing (22). The frequency of oral lesions in trotters were found to be higher (22) than those in event horses examined after a cross-country test (21). This difference is surprising given the relatively high speed in both event and harness racing horses and the associated challenges to steer the horse toward fences and around the racetrack, respectively, while running fast. The difference in frequency of oral lesions is particularly striking for severe lesions, which were present in 20% of trotters but only 4% of event horses (21). It may be that the harmful impact of sustained bit pressures in habituating horses to bit cues are feared more by event riders than by trotting drivers and that, accordingly, the event riders are more moderate in their use of rein tension and more likely to provide partial release. The levels of rein tension discovered in the current study were considerably higher than previous reports in riding horses. Despite that, oral lesions in the area of the bit were not found in the horses before participating in the study, despite that they were in regular training. This suggests that other factors may cause such lesions, in addition to excessive rein tension levels *per se*. For example, all the horses in the study wore a single-jointed snaffle driving bit, which has been reported to be associated with lower risk of oral injuries after a race compared to a Crescendo bit, a mullen mouth bit or a straight plastic bit (28). Additionally, no overcheck was used, whereas this is commonly used in races. Further studies on the relationships among rein tension levels, oral injuries, and equipment use would be valuable.

### Rein tension by segment

When interpreting differences between exercise test segments, the design of the current study needs to be considered. The exercise test comprised walk from the stables to the track, warm-up at moderate trotting speed, then two bouts of fast (racing) trot, first one bout around the track to the left followed by one bout to the right, and finally walk back to the stables. Each bout of fast trot began by circling the horses in the opposite direction, i.e., circling right before going in left direction around the track, and *vice versa*. In Sweden, this “circular volt start” method is common for starting harness races, along with starts behind a moving starting gate. The study horses were accustomed to circling to the right and racing in left direction around the track. While the horses were also accustomed to traveling to the right around the track, as this is the usual direction for warm-up, circling to the left was less familiar to them.

When traveling to the left around the track (the direction the horses were trained to race in), median rein tension was lower during the initial speed-up. However, segment median as well as maximum rein tension were higher during fast trot and slowing when traveling to the left than to the right. During slow trotting and the transition to walk afterwards, segment maximum rein tension was higher when traveling to the right, but no difference in the median rein tension was found. These inconsistent differences between left and right direction likely reflect the combined influence of several factors. The reason for higher rein tension when trotting fast to the left may be because the horses associated this direction with racing. The fact that this was the first bout in all trials may also have contributed to this finding. For comparison, there was a tendency toward decreased rein tension during the second and third tests of the day, compared to the first (*p* = 0.07 for the main effect of repetition). Based on the high levels of rein tension during slowing down to the left, it appears that the horses were unwilling to stop, and therefore perhaps eager to accelerate again. The reason for higher tension during the initial speed-up to the right might then represent a carryover effect from this. The ease or difficulty of circling in one direction rather than the other was likely not a major contributing factor because, for a representative sample of data limited to circling, back-transformed least square means were 22 N for both directions.

The segment interquartile range was used as an indicator for the relative consistency of or variation in rein tension. Interquartile range least square means were largest during the circular volte start and initial speed-up (28–31 N), followed by slowing-down segments (12–18 N). The other segments showed lower values (5–9 N for walk and 8–10 N for fast trot). From the premise that rein tension tends to vary with speed, it makes sense that rein tension would be more variable during the segments that involved speed changes. Reviewing raw data graphs from the slowing-down segments (data not shown), typical measurements showed a pattern with a few seconds of higher tension followed by a few seconds with lower (releasing) tension, which repeated a few times. Thus, this seems to be a common strategy used for slowing-down from racing speed to slow trot.

Both repetition number and position in the field were non-significant in all of the three models, i.e., for segment median, maximum and interquartile range rein tension. However, when position in the field was modeled as an interaction with segment, maximum rein tension was significantly higher at racing trot for the horse in third position, than for the horse in second position. While this finding should be interpreted with some care, it may reflect that the third horse needed to be restrained more to remain in place behind the leader, whereas the horse on the outside perhaps needed fewer deceleratory cues as this horse has to travel a longer distance through the curves. The position outside the leader is subjectively perceived by drivers to be a more strenuous position for the horse.

### Hind limb asymmetry

Above threshold movement asymmetries at trot, potentially indicative of lameness, are very common both in riding horses (29) and in young trotters (30–32) considered sound by their owners. The relationship between motion symmetry and performance appears relatively weak, but horses that showed the poorest lifetime race activity tended to show elevated hind limb asymmetry (32). In the current study, if the horse was hind limb asymmetric, this was associated with substantially increased median rein tension, from 28 N to 48 N. Similarly, maximum values increased from 71 N to 104 N. The immediate cause of this relationship remains unclear, but perhaps horses with a more unstable gait have an increased tendency to seek support by hanging on the bit. This is indeed a common notion among drivers, and markedly uneven rein tension is often perceived as a sign of (subclinical) lameness. That said, asymmetry was evaluated on a single occasion before the trials. It would have been valuable to measure the horses' movements during the exercise tests as well to investigate immediate associations between rein tension and asymmetry on a stride-by-stride basis. Recently published data showed that asymmetries recorded in-hand and when Standardbred yearlings were driven on the track did not differ significantly (31). Moreover, results from Starke et al. (33) revealed no significant changes in asymmetry based on speed of trotting horses in hand on a straight line. However, speed needs to be considered in future studies as it is not known whether the correlations between, e.g., trotting in hand and high speed trotting on a racetrack as compared to slower trot remain robust. Nevertheless, considering these findings, combining rein tension measurements on track and evaluation of movement patterns in hand may still be sufficient to detect relevant associations. More importantly, the horses with movement asymmetries in the current study were few, with three of the nine horses labeled as hind limb asymmetric. The suggested relationship between rein tension and motion asymmetry therefore needs to be verified in another study using a large sample of horses from different age groups, and repeated monitoring of the same horse over time.

### Heart rate

That rein tension increased with increasing heart rate was expected because heart rate increases with the muscular effort associated with increased speed. Previous studies have confirmed that faster gaits are generally associated with higher rein tension (10, 11). Intuitively, where rein tension is being used to restrain horses, one might expect that, if horses perceived the increased workload as onerous, they would require less restraint. However, since the effect of heart rate was evaluated across all segments, and both heart rate and speed varied systematically between segments, the current data do not allow to differentiate between associations of speed and rein tension from those between heart rate and rein tension. Fundamentally, the current study was not designed to address the effects of speed vs. exercise intensity or fatigue on rein tension but these potential relationships merit exploration in future studies.

### Differences among drivers and horses

The significant between-driver differences arose largely from the data on two of the 11 drivers, who stood out from the rest of the group. Driver 7 had the highest estimated rein tension, while Driver 8 had the smallest magnitude of rein tension as compared to the remaining drivers. There was no indication that rein tension differed between male and female drivers. Comparing between horses, Horse 3 (a mare) had the highest rein tension, which differed significantly from most other horses. When evaluated in subject-specific models, four of the nine horses showed significant left-right differences in median rein tension, but none of the drivers did. Of the three horses with hind limb asymmetry, two of them showed a significant difference between left and right rein tension. This may reflect inherent laterality (34) or other forms of asymmetry as these horses were not diagnosed as lame when visually assessed by an experienced equine veterinarian and were perceived to perform normally during training. However, it is not possible to completely rule out low-grade pain or subclinical lameness as a cause, as those conditions can be difficult to diagnose. It is perhaps surprising that drivers, despite reporting a left or right hand preference for manual tasks (handedness), were able to maintain a relatively even rein tension, as indicated by the absence of significant left-right differences. In general, the horse seems to influence rein tension levels more strongly than the rider or driver. Variation between horses constituted a larger fraction of the total random variation in the mixed models on all data, compared to drivers. The same finding has previously been reported in riding horses (2, 35). This does not mean that the horse is solely responsible for the rein tension, but suggests that, with some horses, it is more difficult for the rider or driver to maintain low as well as similar levels of rein tension, which could be due to the horse's innate or acquired tendency to accelerate spontaneously. It would be interesting to further investigate if there are identifiable reasons for such difficulties with certain horses, in which case there is a potential for drivers and riders to further educate the horses and themselves on how to minimize any such factors. Also, there are recent studies of leash tension in dogs that suggest that tri-axial accelerometry can reveal the relative contributions to leash tension from humans and animal members of the dyad (36). It would be interesting to explore if the same technology can be used to decipher some horse-rider/driver interactions.

### Limitations and benefits

The current equipment and procedures deployed for measuring rein tension led to some incomplete data traces. One possible reason for this data loss is that the Bluetooth signal was obstructed by the horses' body as the mobile phone was carried in the driver's pocket. Because of the data loss, where rein tension peaks could not be fully identified, stride cycle related variations were only partially captured. Therefore, we decided to filter the data to estimate stride-median rein tension, which practically eliminated the stride-to-stride pattern (Figure 1). Data sequences were selected from a manual time protocol based on real-time observation of the experiment by a single person, this potentially rendered the data segmentation rather imprecise (Figure 2). Concurrent heart rate and speed recordings were helpful in delineating the exercise test segments in the data, but the equipment occasionally failed to record accurate heart rate data throughout registration. Also, circling events were not timed specifically, resulting in that segment containing both circling and some subsequent acceleration. Differences between left and right track direction can be evaluated by comparing corresponding exercise test segments. However, because left-right direction was consistent rather than randomized, the observed differences may include the order effect of the first vs. the second bout of fast trot. Accordingly, the left and right direction comparisons reported here should be interpreted with care.

## Conclusion

Using a tailored exercise test, rein tension during harness trotting varied substantially among exercise test segments and was generally higher than has been reported during riding. Consistent left-right asymmetry in rein tension appears more common among horses than among drivers. Hind limb asymmetry was associated with increased rein tension, which should be further evaluated in a larger group of horses. The comparably high rein tension in harness trotting, together with the existing reports of a high prevalence of oral injuries among trotters, suggest the need for further research into relationships among rein tension, motion symmetry, use of equipment, and the quality of horse-driver communication.

## Data availability statement

The original contributions presented in the current study are included in the article. Additional data that support the findings of this study can be found in the Supplementary material and via figshare at this link https://doi.org/10.6084/m9.figshare.21195133.v1. Further inquiries can be directed to the corresponding author.

## Ethics statement

The animal study was reviewed and approved by Umeå Local Animal Ethics Committee, Sweden (Protocol A 18-2021).

## Author contributions

EH took a leading role in applying for grant money from the Swedish-Norwegian Foundation for Equine Research. EH, KK-H, MP, MC, and MK conducted the experimental part at Wången. AE, AB, and MK have been preparing the data for analysis. AE and AB have run the statistical analysis and written up the manuscript. All authors have been actively involved in conceptualizing the project idea, discussing the experimental setup, and in reviewing the manuscript.

## Funding

This work was supported by the Swedish-Norwegian Foundation for Equine Research (Stiftelsen Hästforskning) under the Grant Number H-20-47-562.

## Conflict of interest

The authors declare that the research was conducted in the absence of any commercial or financial relationships that could be construed as a potential conflict of interest.

## Publisher's note

All claims expressed in this article are solely those of the authors and do not necessarily represent those of their affiliated organizations, or those of the publisher, the editors and the reviewers. Any product that may be evaluated in this article, or claim that may be made by its manufacturer, is not guaranteed or endorsed by the publisher.

## Supplementary material

Supplementary Figure 1 Median rein tension analysis from joined model, i.e., all subjects [drivers (*n* = 11) or horses (*n* = 9)] estimated as fixed effects (third column and color marking) and subject-specific models (all other columns) to study the difference between left and right rein tension within subjects. For example, three of the horses showed significantly increased rein tension in the left rein and one horse in the right rein. Colored cells in the comparison columns show statistical comparisons performed, green indicates significant statistical difference at *p* < 0.05 while black indicates *p* ≥ 0.05.

Supplementary Table 1 Asymmetry parameters evaluated with the Lameness Locator (IMU-based gait analysis system Equinosis, Columbia, MO, USA) prior to exercise and the interpretation of the results. Horses were classified as hind limb asymmetric (yes = 1, no = 0) if PDmin or PDmax mean was >3 mm and the mean value was lower than the corresponding SD; and classified as forelimb asymmetric if HDmin mean was >6 mm and lower than SD.

## References

[1] McGreevy PD. The advent of equitation science. Vet J. (2007) 174:492–500. 10.1016/j.tvjl.2006.09.008 17157542

[2] Eisersiö M Rhodin M Roepstorff L Egenvall A. Rein tension in 8 professional riders during regular training sessions. J Vet Behav. (2015) 10:419–26. 10.1016/j.jveb.2015.05.004

[3] Egenvall A Clayton HM Eisersiö M Roepstorff L Byström A. Rein tension in transitions and halts during equestrian dressage training. Animals. (2019) 9:712. 10.3390/ani9100712 31547540 PMC6827353

[4] Fenner K Freire R McLean A McGreevy P. Behavioral, demographic, and management influences on equine responses to negative reinforcement. J Vet Behav. (2019) 29:11–7. 10.1016/j.jveb.2018.08.007

[5] Eisersiö M Yngvesson J Byström A Baragli P Egenvall A. A rein tension signal can be reduced by half in a single training session. Appl Anim Behav Sci. (2021) 243:105452. 10.1016/j.applanim.2021.105452

[6] Warren-Smith AK Curtis RA Greetham L McGreevy PD. Rein contact between horse and handler during specific equitation movements. Appl Anim Behav Sci. (2007) 108:157–69. 10.1016/j.applanim.2006.11.017

[7] Kau S Potz IK Pospisil K Sellke L Schramel JP Peham C. Bit type exerts an influence on self-controlled rein tension in unridden horses. Sci Rep. (2020) 10:1–13. 10.1038/s41598-020-59400-w 32051498 PMC7016124

[8] Piccolo L Kienapfel K. Voluntary rein tension in horses when moving unridden in a dressage frame compared with ridden tests of the same horses—a pilot study. Animals. (2019) 9:321. 10.3390/ani9060321 31174265 PMC6616402

[9] Preuschoft H Witte H Recknagel S Bär H Lesch C Wüthrich M. Über die Wirkung gebräuchlicher Zäumungen auf das Pferd. Dtsch Tieratzliche Wochenschrift. (1999) 106:167–75. 10354650

[10] Dumbell L Lemon C Williams J. A systematic literature review to evaluate the tools and methods used to measure rein tension. J Vet Behav. (2019) 29:77–87. 10.1016/j.jveb.2018.04.003

[11] Clayton H MacKechnie-Guire R Byström A Le Jeune S Egenvall A. Guidelines for the measurement of rein tension in equestrian sport. Animals. (2021) 11:2875. 10.3390/ani11102875 34679895 PMC8532849

[12] Egenvall A Roepstorff L Eisersiö M Rhodin M van Weeren R. Stride-related rein tension patterns in walk and trot in the ridden horse. Acta Vet Scand. (2015) 57:1–10. 10.1186/s13028-015-0182-3 26715156 PMC4696263

[13] Clayton H Singleton W Lanovaz J Cloud G. Measurement of rein tension during horseback riding using strain gage transducers. Exp Tech. (2003) 27:34–6. 10.1111/j.1747-1567.2003.tb00112.x

[14] Byström A Rhodin M Von Peinen K Weishaupt MA Roepstorff L. Basic kinematics of the saddle and rider in high-level dressage horses trotting on a treadmill. Equine Vet J. (2009) 41:280–4. 10.2746/042516409X394454 19469236

[15] Parkes RS Weller R Pfau T Witte TH. The effect of training on stride duration in a cohort of two-year-old and three-year-old thoroughbred racehorses. Animals. (2019) 9:466. 10.3390/ani9070466 31336595 PMC6680649

[16] Tell A Egenvall A Lundström T Wattle O. The prevalence of oral ulceration in Swedish horses when ridden with bit and bridle and when unridden. Vet J. (2008) 178:405–10. 10.1016/j.tvjl.2008.09.020 19027332

[17] Björnsdóttir S Frey R Kristjansson T Lundström T. Bit-related lesions in Icelandic competition horses. Acta Vet Scand. (2014) 56:1–7. 10.1186/s13028-014-0040-8 25116656 PMC4236600

[18] Mata F Johnson C Bishop C. A cross-sectional epidemiological study of prevalence and severity of bit-induced oral trauma in polo ponies and race horses. J Appl Anim Welfare Sci. (2015) 18:259–68. 10.1080/10888705.2015.1004407 25679445

[19] Mellor DJ. Mouth pain in horses: physiological foundations, behavioural indices, welfare implications, and a suggested solution. Animals. (2020) 10:572. 10.3390/ani10040572 32235343 PMC7222381

[20] Uldahl M Clayton H. Lesions associated with the use of bits, nosebands, spurs and whips in Danish competition horses. Equine Vet J. (2019) 51:154–62. 10.1111/evj.12827 29502345

[21] Tuomola K Maki-Kihnia N Valros A Mykkanen A Kujala-Wirth M. Bit-related lesions in event horses after a cross-country test. Front Vet Sci. (2021) 8:651160. 10.3389/fvets.2021.651160 33869325 PMC8044447

[22] Tuomola K Mäki-Kihniä N Kujala-Wirth M Mykkänen A Valros A. Oral lesions in the bit area in finnish trotters after a race: lesion evaluation, scoring, and occurrence. Front Vet Sci. (2019) 6:206. 10.3389/fvets.2019.00206 31355213 PMC6640207

[23] Hartmann E Byström A Pökelmann M Connysson M Kienapfel-Henseleit K Karlsteen M. Associations between driving rein tensions and drivers' reports of the behaviour and driveability of Standardbred trotters. Appl Anim Behav Sci. (2022) 105726. 10.1016/j.applanim.2022.105726

[24] Keegan KG Kramer J Yonezawa Y Maki H Pai PF Dent EV. Assessment of repeatability of a wireless, inertial sensor-based lameness evaluation system for horses. Am J Vet Res. (2011) 72:1156–63. 10.2460/ajvr.72.9.1156 21879972

[25] Egenvall A Roepstorff L Rhodin M Eisersiö M Clayton HM. Maximum and minimum peaks in rein tension within canter strides. J Vet Behav. (2016) 13:63–71. 10.1016/j.jveb.2016.03.007

[26] Bogisch S Geser-von Peinen K Wiestner T Roepstorff L Weishaupt MA. Influence of velocity on horse and rider movement and resulting saddle forces at walk and trot. Compa Exer Physiol. (2014) 10:23–32. 10.3920/CEP13025

[27] Weishaupt MA Hogg H Auer J Wiestner T. Velocity-dependent changes of time, force and spatial parameters in Warmblood horses walking and trotting on a treadmill. Equine Vet J. (2010) 42:530–7. 10.1111/j.2042-3306.2010.00190.x 21059056

[28] Tuomola K Mäki-Kihniä N Valros A Mykkänen A Kujala-Wirth M. Risk factors for bit-related lesions in Finnish trotting horses. Equine Vet J. (2021) 53:1132–40. 10.1111/evj.13401 33336423 PMC8518388

[29] Rhodin M Egenvall A Haubro Andersen P Pfau T. Head and pelvic movement asymmetries at trot in riding horses in training and perceived as free from lameness by the owner. PLoS ONE. (2017) 12:e0176253. 10.1371/journal.pone.0176253 28441406 PMC5404851

[30] Ringmark S Jansson A Lindholm A Hedenström U Roepstorff L. A 2.5 year study on health and locomotion symmetry in young Standardbred horses subjected to two levels of high intensity training distance. Vet J. (2016) 207:99–104. 10.1016/j.tvjl.2015.10.052 26654845

[31] Kallerud AS Fjordbakk CT Hendrickson EH Persson-Sjodin E Hammarberg M Rhodin M. Objectively measured movement asymmetry in yearling Standardbred trotters. Equine Vet J. (2021) 53:590–9. 10.1111/evj.13302 32558997

[32] Jansson A Ringmark S Johansson L Roepstorff L. Locomotion asymmetry in young Standardbred trotters in training and links to future racing career. Comp Exerc Physiol. (2022) 18:85–92. 10.3920/CEP210035

[33] Starke SD Raistrick KJ May SA Pfau T. The effect of trotting speed on the evaluation of subtle lameness in horses. Vet J. (2013) 197:245–52. 10.1016/j.tvjl.2013.03.006 23611486

[34] Byström A Clayton H Hernlund E Rhodin M Egenvall A. Equestrian and biomechanical perspectives on laterality in the horse. Comp Exerc Physiol. (2020) 16:35–45. 10.3920/CEP190022

[35] Kuhnke S Dumbell L Gauly M Johnson JL McDonald K Von Borstel UK. A comparison of rein tension of the rider's dominant and non-dominant hand and the influence of the horse's laterality. Comp Exerc Physiol. (2010) 7:57–63. 10.1017/S1755254010000243

[36] Shih H-Y Georgiou F Curtis RA Paterson M Phillips CJ. Behavioural evaluation of a leash tension meter which measures pull direction and force during human–dog on-leash walks. Animals. (2020) 10:1382. 10.3390/ani10081382 32785117 PMC7460501

