# Supplementary figures and images for: Rein tension in harness trotters during on-track exercise
Source: Front Vet Sci. 2022 Oct 11;9:987852. doi: 10.3389/fvets.2022.987852 (PMC9592803; doi:10.3389/fvets.2022.987852)

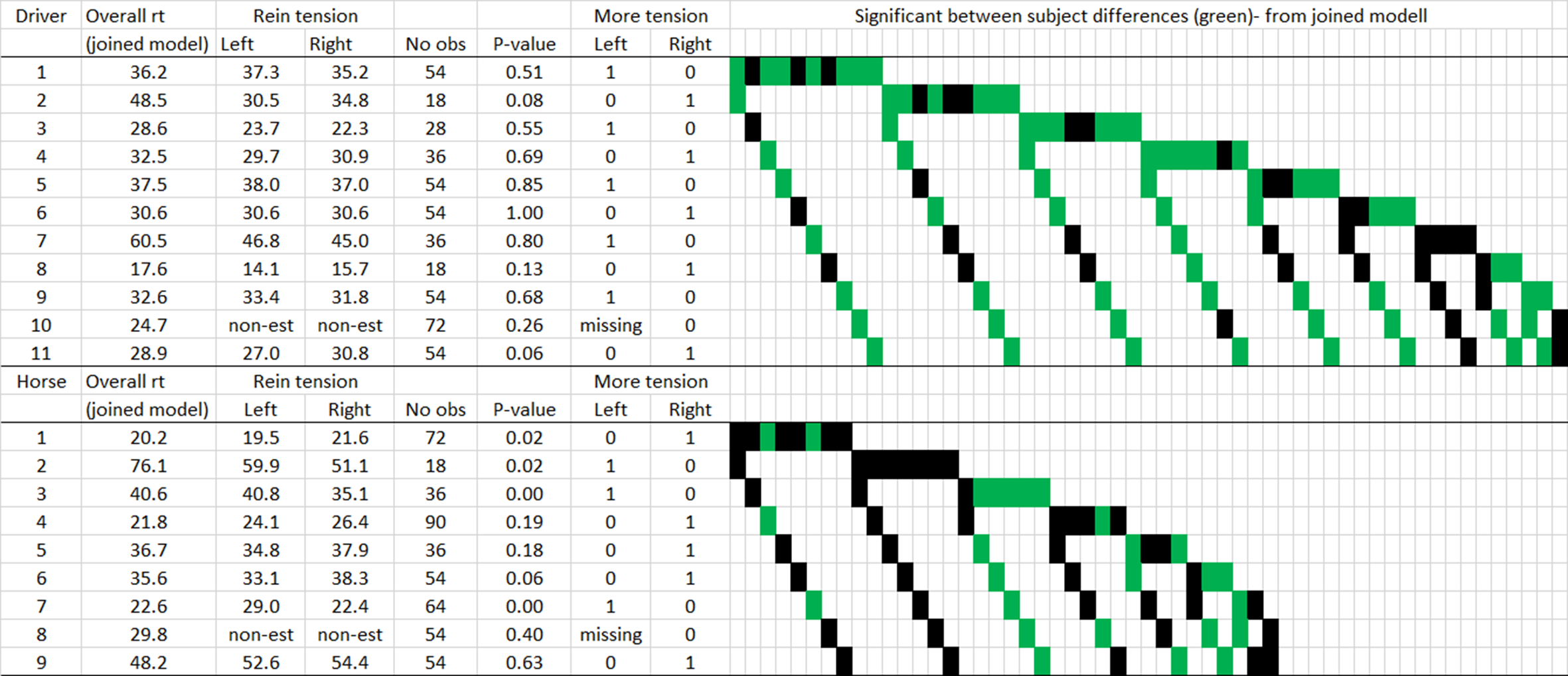

Supplement: Supplementary Figure 1 — Median rein tension analysis from joined model, i.e., all subjects [drivers (n = 11) or horses (n = 9)] estimated as fixed effects (third column and color marking) and subject-specific models (all other columns) to study the difference between left and right rein tension within subjects. For example, three of the horses showed significantly increased rein tension in the left rein and one horse in the right rein. Colored cells in the comparison columns show statistical comparisons performed, green indicates significant statistical difference at p < 0.05 while black indicates p ≥ 0.05. [file Image_1.tif]
