# Supplementary material for: Rein tension in harness trotters during on-track exercise
Source: Front Vet Sci. 2022 Oct 11;9:987852. doi: 10.3389/fvets.2022.987852 (PMC9592803; doi:10.3389/fvets.2022.987852)
Supplement: Supplementary Table 1 — Asymmetry parameters evaluated with the Lameness Locator (IMU-based gait analysis system Equinosis, Columbia, MO, USA) prior to exercise and the interpretation of the results. Horses were classified as hind limb asymmetric (yes = 1, no = 0) if PDmin or PDmax mean was >3 mm and the mean value was lower than the corresponding SD; and classified as forelimb asymmetric if HDmin mean was >6 mm and lower than SD. [file Table_1.docx]

|  | HDmin^1^ | | HDmax^2^ | | PDmin^3^ | | PDmax^4^ | | Asymmetric | | Number of strides | |
| --- | --- | --- | --- | --- | --- | --- | --- | --- | --- | --- | --- | --- |
| Horse | Mean | SD | Mean | SD | Mean | SD | Mean | SD | forelimb | hind limb | forelimb | hind limb |
| 1 | -10.8 | (15.5) | 8 | (17.0) | 5.6 | (11.6) | 2.3 | (9.4) | 0 | 0 | 23 | 23 |
| 2 | 16.5 | (9.9) | 9.7 | (20.5) | -8.9 | (8.7) | -0.3 | (6.6) | 1 | 1 | 18 | 23 |
| 3 | -6.5 | (11.1) | -4.7 | (11.6) | -6.8 | (2.2) | -6.1 | (4.3) | 0 | 1 | 18 | 23 |
| 4 | -0.2 | (15.2) | 3.5 | (16.4) | -2.1 | (6.3) | -3.1 | (4.5) | 0 | 0 | 24 | 23 |
| 5 | -11.8 | (23.4) | -0.1 | (18.8) | -9.2 | (10.8) | -4.4 | (8.4) | 0 | 0 | 15 | 23 |
| 6 | -7.1 | (13.2) | -21.4 | (13.2) | -1.1 | (7.3) | -0.8 | (4.7) | 1 | 0 | 21 | 23 |
| 7 | 0.5 | (10.5) | 1.7 | (14.0) | -0.6 | (8.7) | 0.8 | (5.5) | 0 | 0 | 21 | 23 |
| 8 | 9.4 | (15.2) | -1.7 | (18.1) | 3.9 | (7.1) | 1.5 | (4.5) | 0 | 0 | 24 | 23 |
| 9 | 6.5 | (10.2) | 4.8 | (11.9) | 12.4 | (6.2) | 7.6 | (6.8) | 0 | 1 | 19 | 23 |
| ^1^HDmin=difference vertical displacement minima for poll between right and left stance | | | | | | | | | | | | |
| ^2^HDmax= difference vertical displacement maxima for poll between right and left stance | | | | | | | | | | | | |
| ^3^PDmin= difference vertical displacement minima for pelvis between right and left stance | | | | | | | | | | | | |
| ^4^PDmax= difference vertical displacement maxima for pelvis between right and left stance | | | | | | | | | | | | |

**Supplementary Table 1.**

Asymmetry parameters evaluated with the Lameness Locator (IMU-based gait analysis system Equinosis, Columbia, MO, USA) prior to exercise and the interpretation of the results. Horses were classified as hind limb asymmetric (yes = 1, no = 0) if PDmin or PDmax mean was >3 mm and the mean value was lower than the corresponding SD; and classified as forelimb asymmetric if HDmin mean was > 6 mm and lower than SD.
